# Avoiding False Identification of 7‑Hydroxymitragynine in Kratom Products Using a Multicriteria LC–MS Confirmation

**DOI:** 10.1021/jasms.6c00088

**Published:** 2026-04-16

**Authors:** Daniel Sheehan, Yanfang Li, Emily Meckler, Roy Upton, Mengliang Zhang

**Affiliations:** 1 Department of Chemistry and Biochemistry, 1354 Ohio University, Athens, Ohio 45701, United States; 2 535553 American Herbal Pharmacopoeia, Scotts Valley, California 95066, United States

**Keywords:** kratom, LC−MS, mitragynine, 7-hydroxymitragynine, mitragynine pseudoindoxyl

## Abstract

Kratom (*Mitragyna specios*a) products have become widely available in diverse commercial formulations, raising increasing analytical and regulatory interest due to the presence of potent alkaloids such as mitragynine (MG), 7-hydroxymitragynine (7-OH), and mitragynine pseudoindoxyl (MGP). The accurate identification of these compounds is analytically challenging because kratom matrices contain numerous structurally related alkaloids and isomeric species that produce similar mass spectral features. In this study, an ultrahigh-performance liquid chromatography–high-resolution mass spectrometry (UHPLC–HRMS) workflow was developed to characterize kratom alkaloids in authentic leaf materials and 38 commercial kratom products representing multiple formulations. Chromatographic separation combined with accurate mass measurement and diagnostic MS/MS fragmentation enabled reliable differentiation of MG, 7-OH, MGP, and related isomers. Multiple overlapping chromatographic peaks were observed at *m*/*z* 415.2227, demonstrating that reliance solely on accurate mass or commonly monitored transitions such as *m*/*z* 415 → 190 can lead to false-positive identification of 7-OH in complex botanical matrices. Application of the multicriteria confirmation strategy revealed substantial variability in alkaloid composition across commercial products, including the presence of 7-OH and MGP in several tablet and capsule formulations. Untargeted metabolomic profiling further distinguished kratom plant extracts, including the authentic leaf materials, from processed products enriched in specific alkaloids. These results highlight the importance of integrating chromatographic resolution with orthogonal MS/MS criteria for reliable identification of kratom alkaloids and provide an analytical framework for the characterization of complex botanical products.

## Graphical abstract

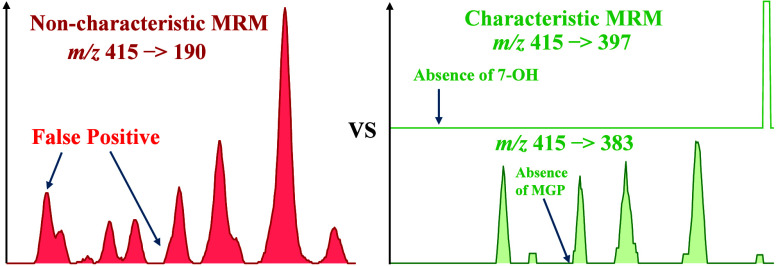

## Introduction

Kratom (*Mitragyna speciosa*) has rapidly emerged as a widely consumed psychoactive product in the United States. However, it is not approved by the U.S. Food and Drug Administration (FDA) as a drug, dietary supplement, or food ingredient,^1^ and its regulatory status remains complex and evolving. Although kratom is not federally scheduled, several U.S. states, including Alabama, Arkansas, Indiana, Rhode Island, and Wisconsin, have enacted full bans, and additional jurisdictions have pending legislation to restrict or prohibit its sale and use. At the federal level, the FDA has issued multiple warnings regarding kratom’s safety and has expressed intent to restrict or prohibit products containing 7-hydroxymitragynine (7-OH) due to toxicity concerns.^2,3^ In December 2025, the U.S. Marshals, acting in coordination with the FDA, seized approximately 73,000 units of 7-OH products, including tablets, liquid shots, and gummies, valued at nearly $1 million, from firms in Missouri.^4^ Meanwhile, the Drug Enforcement Administration (DEA) previously announced its intent to place kratom’s major alkaloids, mitragynine and 7-OH, into Schedule I.^5^ These regulatory uncertainties underscore the need for rigorous chemical characterization and analytical surveillance of kratom products currently available in the U.S. marketplace.

Compounding the regulatory uncertainty is the rapid expansion, diversification, and accessibility of kratom products to consumers. Kratom is sold in numerous forms, including live plants, raw plant powders, capsules, tablets, resin extracts, concentrated liquids, gummies, vape liquids, and “enhanced” or “extract” products, including those containing semisynthetic derivatives of 7-OH. These items are readily accessible both in-person and online, commonly sold in gas stations, smoke shops, convenience stores, and through e-commerce retailers with minimal to no age restrictions.^6^ Many products are marketed as “natural,” “herbal,” or “wellness” supplements despite lacking standardization, quality control, or regulatory oversight. This variability increases the likelihood of exposure to highly heterogeneous and potentially modified products, presenting challenges for forensic analysis, toxicological interpretation, and regulatory monitoring. Accurate identification of kratom alkaloids within these complex matrices remains analytically challenging due to the presence of structurally similar and isomeric compounds.

Kratom’s growing popularity is largely driven by perceptions of therapeutic benefit and in part due to its promotion as a natural psychoactive agent. Mitragynine, the most abundant natural alkaloid in kratom leaves, is currently under active investigation as an atypical opioid with reduced respiratory depression and gastrointestinal side effects compared to classical opioids.^7–9^ Early data suggest that mitragynine-based therapeutics may offer safer alternatives for pain management and opioid withdrawal, with the potential to improve treatment adherence and completion rates in opioid detoxification programs.^9^ At the community level, kratom is widely used informally to self-manage opioid withdrawal, reduce opioid consumption, and alleviate dependence symptoms. Among polydrug users, kratom use has also been associated with reduced methamphetamine use, supporting its potential harm-reduction value. Conversely, adverse effects, including death, have been reported for kratom alone as well as its use with other addictive substances, most notably conventional opioids.^10^ Of increasing concern is the emergence of semisynthetic and chemically modified kratom products. While natural kratom leaves contain no or very low levels of 7-OH^11^ and virtually no detectable mitragynine pseudoindoxyl (MGP), some commercial “extract,” “enhanced,” or tablet-based products contain high concentrations of these compounds.^12–14^ These molecules exhibit substantially greater μ-opioid receptor potency, with MGP producing marked respiratory depression comparable to morphine. Such potency differences highlight critical pharmacological distinctions between natural kratom alkaloids and semisynthetic derivatives, which increase the risk of overdose, dependency, and toxicity associated with highly concentrated kratom products. Consumers purchasing products labeled as “kratom” or “7-OH kratom” may unknowingly ingest substances with pharmacological effects more closely aligned with potent synthetic opioids than with natural kratom. This discrepancy raises substantial concerns for consumer safety, misbranding, overdose risk, and toxicological interpretation. It also highlights a pressing regulatory problem: chemical modifications can fundamentally alter a product’s risk profile, yet these products remain available on the commercial market with inconsistent and unclear enforceable standards.

Given the complexity of modern kratom products, robust analytical tools are essential for accurate identification, quantification, and differentiation of natural versus semisynthetic constituents. Multiple kratom alkaloids share structural features, stereochemistry, and fragmentation pathways, complicating mass spectral interpretation and quantification.^15–17^ Diastereomeric and positional isomers, such as mitragynine, speciociliatine, speciogynine, and mitraciliatine, require chromatographic resolution for accurate identification. Liquid chromatography–mass spectrometry (LC–MS) has emerged as a reliable analytical platform for kratom alkaloid characterization owing to its high sensitivity, selectivity, and ability to resolve structurally similar indole alkaloids. LC–MS enables simultaneous identification and quantification of multiple kratom alkaloids, detection of semisynthetic analogues, and confirmation of product authenticity, making it indispensable for forensic casework, toxicology, regulatory monitoring, and product standardization.^17^ However, accurate identification of kratom alkaloids remains analytically challenging because many indole alkaloids share similar or exact masses and fragmentation pathways. Despite widespread use, commonly monitored transitions such as *m*/*z* 415 → 190 for 7-OH or MGP lack structural specificity and may lead to false-positive identification in complex botanical matrices. To date, this limitation has not been systematically demonstrated. In this study, we provide direct chromatographic and MS/MS evidence of such misidentification and propose a multicriteria confirmation strategy to overcome this limitation.

We surveyed chemical profiles of 38 commercial kratom products, including plant powder, powder extract, capsules, tablets, gummies, and drink mix, and compared them with authentic kratom leaves using LC–MS. We identified the critical identification challenges for 7-OH and MGP and proposed a comprehensive approach to confidently confirm the identities of both highly concerning compounds in the commercial kratom products. Lastly, we describe the inclusion of multiple compound features for reliably distinguishing naturally sourced kratom products from chemically modified kratom products. The proposed identification framework relies on chromatographic resolution and diagnostic MS/MS fragments, making the approach applicable to a wide range of LC–MS platforms used in routine testing laboratories.

## Materials and Methods

### Chemicals and Materials

LC–MS grade acetonitrile (ACN), methanol, formic acid, and HPLC grade water were obtained from Fisher Scientific, Inc. (Fair Lawn, NJ, USA). Mitragynine (MG), 7-hydroxymitragynine (7-OH), and mitragynine pseudoindoxyl (MGP) standards were purchased from Cayman Chemical (Ann Arbor, MI, USA). QuEChERS extract pouch was purchased from Agilent Technologies (Palo Alto, CA, USA).

### Sample Preparation

A total of 39 commercial kratom products were obtained from the U.S. market, including 6 plant powder samples, 6 liquid plant extract samples, 9 tablet samples, 10 capsule samples, 4 gummy samples, 2 soft-gel samples, 1 sparkling water mix, and 1 drink-mix sample. In addition, one botanically authenticated kratom voucher specimen (AHP-Verified) was obtained from the American Herbal Pharmacopoeia (AHP) and is deposited in the herbarium collection of AHP. The details of these products are provided in Table S1. KM35 was a sparkling water mix sample containing kratom that was initially processed using the same preparation protocol as the drink powder samples. However, due to the low alkaloid concentrations in this sample, the applied dilution and extraction conditions were not suitable for reliable detection. Therefore, KM35 was excluded from the study.

Given the distinct physical and chemical characteristics of the matrices, matrix-specific sample-preparation procedures were employed for plant, gummy, tablet, and drink-mix samples, as previously described.^18^ *Plant powder, tablet (crushed to fine powder with a mortar and pestle prior to extraction), powder from capsule, drink-mix and liquid plant extract samples*: One hundred milligrams of the sample were extracted with 10 mL of a 3:7 mixture of water and methanol with 2 mM glacial acetic acid by vortexing for 1 min, followed by vigorous shaking at 300 rpm for 20 min. After settling, 1 mL of the supernatant was filtered through a 0.22-μm nylon syringe filter. *Gummy and soft-gel samples*: One hundred milligrams of the finely chopped sample were extracted with 10 mL of water, followed by the addition of 10 mL of ACN. The mixture was vortexed for 1 min and sonicated for 20 min. A QuEChERS extraction pouch (containing 4 g magnesium sulfate and 1 g sodium chloride) was then added, and the mixture was vortexed for an additional 1 min. After settling for 10 min, the ACN layer was collected and filtered. The samples were diluted 100-fold prior to LC–MS analysis. All samples were prepared and extracted in triplicate.

Unit-to-unit variability was evaluated by extracting 7-OH, MGP, and MG from five individual tablets (KM05, KM06, and KM18) or capsules (KM31 and KM33) (n = 5) using the aforementioned protocol.

### LC–MS Instrumentation

All samples were analyzed using an ultrahigh-performance liquid chromatography–high-resolution mass spectrometry (UHPLC–HRMS) system consisting of a Vanquish UHPLC coupled to an Orbitrap Exploris 120 mass spectrometer (Thermo Scientific, Waltham, MA, USA). Chromatographic separation was performed on a Hypersil GOLD PFP (pentafluorophenyl) HPLC column (2.1 × 100 mm, 1.9 μm; Thermo Scientific, Waltham, MA, USA). The mobile phase consisted of water containing 0.1% formic acid (FA) and 2 mM ammonium acetate (mobile phase A) and methanol containing 0.1% FA and 2 mM ammonium acetate (mobile phase B). The gradient elution program was as follows: 0–0.5 min, 35–45% B; 0.5–3 min, 45% B; 3–10 min, 45 to 80% B; 10–11 min, 80 to 100% B; 11–11.8 min, hold at 100% B; 11.8–12 min, decrease to 35% B; and 12–14 min, hold at 35% B. The column temperature was maintained at 40 °C, with a flow rate of 0.3 mL/min and an injection volume of 2 μL. Full-scan MS and targeted MS^2^ (t-MS^2^) data were acquired using a heated electrospray ionization (H-ESI) source in positive mode over a mass range of *m*/*z* 150–900, with a resolving power of 60,000. The MS source parameters were set as follows: spray voltage, + 3.5 kV; ion transfer tube temperature, 350 °C; vaporizer temperature, 275 °C; sheath gas, 55 arbitrary units (Arb); auxiliary gas, 15 Arb; and sweep gas, 3 Arb. Targeted MS^2^ scans were acquired with a resolution of 15,000 to monitor precursor ions at *m*/*z* 291.0869, 353.1865, 369.1814, 383.1971, 385.2127, 397.2127, 399.2284, 415.2233, and 417.2383 over a 14 min run without retention-time scheduling. These precursor ions were selected to represent key alkaloids and flavonoids commonly observed in kratom samples, including MG, 7-OH/MGP, mitraphylline, paynantheine, rhynchophylline, ajmalicine, corynoxeine, epicatechin, and their isomeric analogs.^20^ Each sample extract was analyzed in triplicate.

### Data Processing

In total, 351 LC–MS data objects were collected (39 samples × 3 extractions × 3 injections = 351). One data object (KM33, Extract No. 1, Injection No. 1) exhibited significant retention time drift due to inadequate HPLC equilibration and was therefore excluded from the data set. The LC–MS raw data (n = 350) were preprocessed with MZmine software (version 4.8.30)^19^ for peak detection, alignment, peak peaking, and integration. Preprocessed data with 2784 features were generated and exported to a CSV file in Excel as a two-dimensional data matrix, including variable indices such as retention time, *m*/*z* (mass-to-charge ratio), peak area, and ion intensity. Potential alkaloids and secondary metabolites precursor ion features, including *m*/*z* 195.0882, 315.2324, 291.0869, 397.2127, 399.2284, 385.2127, 415.2227, 383.1971, 353.1865, and 369.1814, were selected and then used for statistical analysis.^20^ Ions at *m*/*z* 195.0882, 315.2324 were also included to represent caffeine and cannabidiol (CBD), respectively, due to their presence in some kratom samples. Principal component analysis (PCA) score, loading plot, and box plots were generated with MetaboAnalyst 6.0.^21^ The extracted ion chromatogram (EIC) was exported through Xcalibur 4.2 (Thermo Scientific, San Jose, CA, USA) and then plotted using MagicPlot 3.0.1 (Magicplot Systems, LLC, Saint Petersburg, Russia).

## Results and Discussion

### Chromatography Profiles for the Authentic Leaf, 7-OH Tablet, and Gummy Samples

Representative EICs for a 7-OH tablet sample (KM03), a gummy formulation (KM30), and an authentic leaf-cut sample (KM40) are shown in Figure 1. All chromatographic peak intensities were normalized to MG to facilitate comparison of relative alkaloid distributions across matrices.

**Figure 1:**
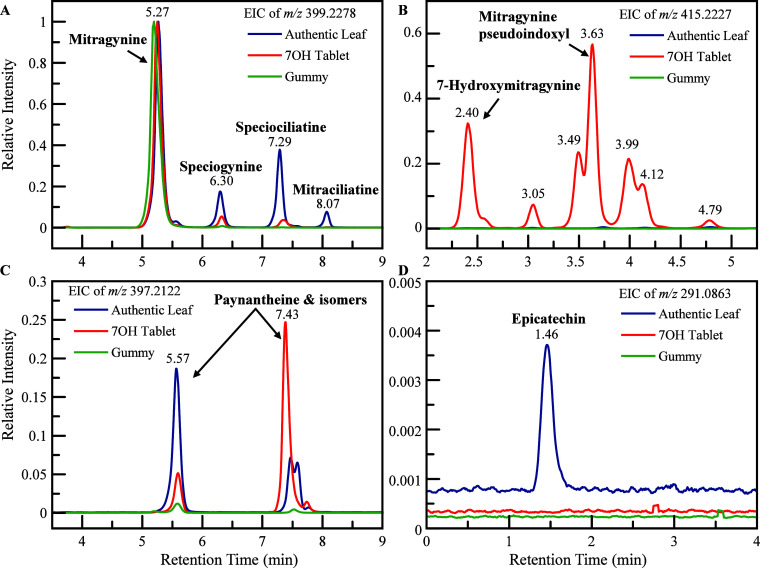
EICs of *m*/*z* 399.2284 (A), *m*/*z* 415.2227 (B), *m*/*z* 397.2122 (C), and *m*/*z* 291.0863 (D) for a representative authentic leaf sample (KM40), 7-OH tablet sample (KM03), and gummy sample (KM30). All chromatographic peak intensities were normalized to the peak height of MG within each sample.

From the EIC of *m*/*z* 399.2284 (for [C_23_H_30_N_2_O_4_+H]^+^, ± 0.001 Da), four alkaloid diastereomers, MG, speciogynine, speciociliatine, and mitraciliatine, were well resolved under the optimized chromatographic conditions (Figure 1A). In authentic leaf samples, speciogynine, speciociliatine, and mitraciliatine were present at substantial relative abundances (∼18%, 40%, and 10% of MG peak height, respectively), consistent with previously reported authentic kratom leaf profiles.^22^ In contrast, the 7-OH tablet product (KM03) exhibited MG as the dominant peak with markedly reduced relative abundances of the other diastereomers, while the gummy sample (KM30) displayed MG as the only detectable compound at this *m*/*z* value. These differences indicate a simplified alkaloid profile in certain processed commercial formulations relative to intact plant material. The chromatographic complexity was more pronounced at *m*/*z* 415.2227 (for [C_23_H_30_N_2_O_5_+H]^+^, ± 0.001 Da) (Figure 1B). The precursor ion at *m*/*z* 415.2227 corresponds to multiple isomeric alkaloids, including 7-OH and MGP, as well as additional structurally related compounds. In the 7-OH tablet extract (KM03), seven distinct chromatographic peaks were detected within the 2–5 min retention window. Using authentic reference standards, two of these peaks were confirmed as 7-OH (2.40 min) and MGP (3.63 min). Notably, a peak at 3.49 min partially coeluted with MGP (3.63 min), highlighting the risk of misidentification in the absence of sufficient chromatographic resolution and orthogonal confirmation. In contrast, neither 7-OH nor MGP was confirmed in the authentic leaf sample (KM40) or the gummy formulation (KM30) under the validated confirmation criteria described in the next section. Although several low-intensity peaks were observed at *m*/*z* 415.2227 in the authentic leaf extract, none matched both the retention time and MS/MS characteristics of authentic 7-OH or MGP (detailed discussion available in the next section and in Figure 2). From the EIC of *m*/*z* 397.2122 (for [C_23_H_28_N_2_O_4_+H]^+^, ± 0.001 Da) (Figure 1C), two major peaks corresponding to paynantheine and related isomers were observed at 5.57 and 7.43 min. Both were present in authentic leaf and commercial products; however, relative intensities differed. The 5.57 min peak was more prominent in authentic leaf samples and less prominent in the 7-OH tablet product, suggesting an alteration in alkaloid distribution associated with processing or formulation. Epicatechin (*m*/*z* 291.0863 for [C_15_H_14_O_6_+H]^+^, error: −2.1 ppm, at 1.46 min) was detected exclusively in the authentic leaf sample (Figure 1D), consistent with the presence of native nonalkaloid phytochemicals in intact plant material and their reduction or removal in processed products.^23^

**Figure 2:**
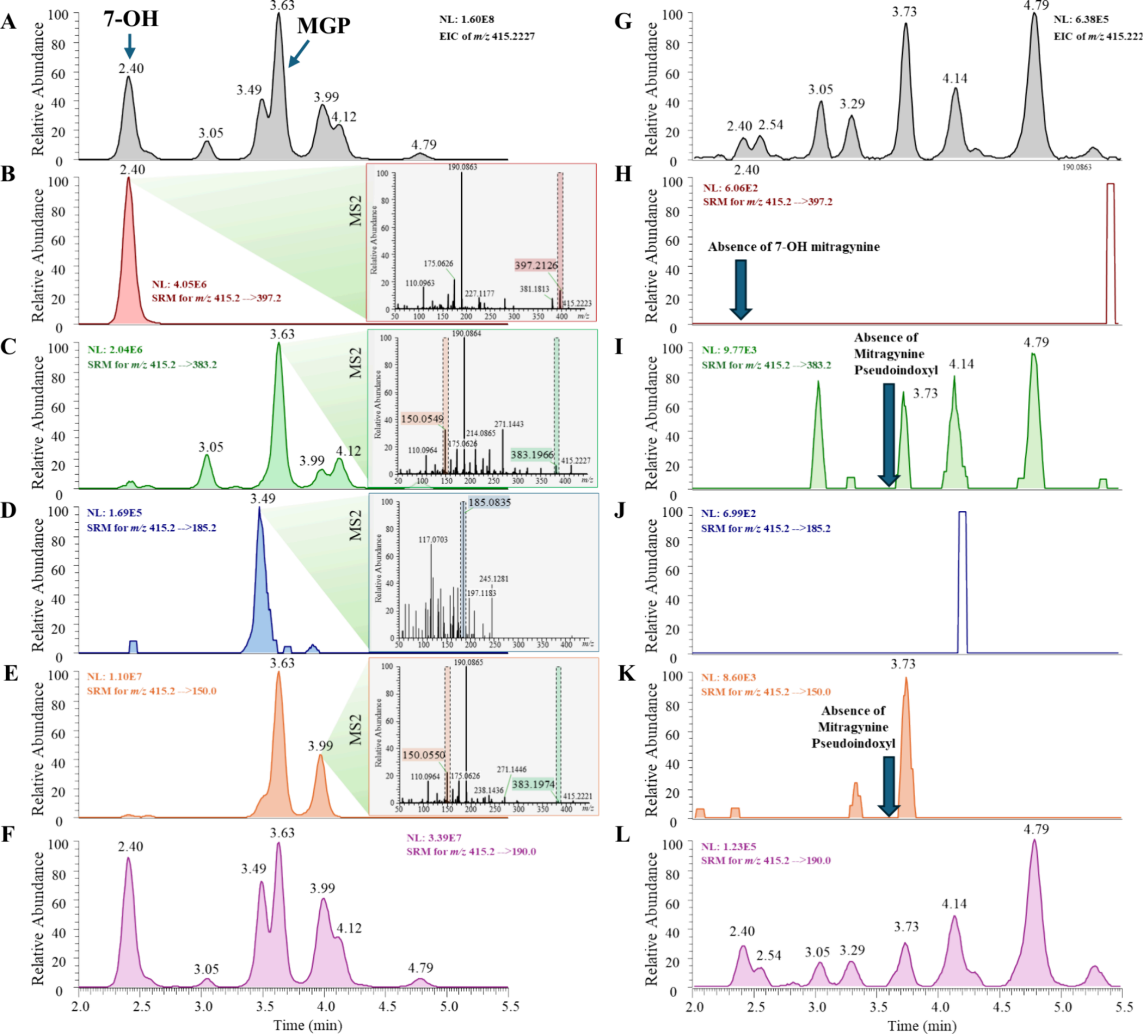
EIC of *m*/*z* 415.2227 for 7-OH/MGP isomers for tablet sample (KM03) (A) and authentic leaf sample (KM40) (G) with their respective selected ion monitoring (SRM) chromatograms in panels B–F and H-L. MS^2^ spectra of 7-OH, MGP, and two isomeric peaks eluting at 3.49 and 3.99 min in the tablet sample (KM03) are embedded in their characteristic SRM chromatograms in panels B, C, D, and E, respectively, with characteristic fragment ions highlighted. Noncharacteristic SRM transition of *m*/*z* 415 → 190 for tablet sample (KM03) is shown in panel F. Corresponding SRM for the authentic leaf sample (KM40) are shown in panels H, I, J, K, and L respectively. All MS^2^ spectra were collected via higher-energy collisional dissociation (HCD) at a normalized collision energy of 50.

Collectively, the metabolite profiles for the authentic leaf cut samples showed strong similarities with the profiles in Figure S1, which was the reassembly of EIC profiles from the raw data of an authentic kratom sample (K-55B) previously published by Cech’s group.^22^ However, substantial variability was observed among the 39 commercial products surveyed, particularly in the distribution and relative abundance of alkaloids at *m*/*z* 415.2227, which will be discussed in detail in the next section.

### Rigorous Confirmation of 7-OH and MGP

Accurate identification of 7-OH and MGP is critical for distinguishing intact botanical material from products potentially containing enriched or semisynthetic constituents. Accurate mass detection at *m*/*z* 415.2227 alone revealed multiple chromatographic peaks in the 7-OH tablet (Figure 2A) and authentic leaf extract samples (Figure 2G), underscoring the complexity of the kratom matrix.

MS/MS spectra were therefore examined for each detected peak. 7-OH exhibited a characteristic fragment ion at *m*/*z* 397.2126 (Figure 2B), corresponding to neutral loss of H_2_O from the protonated molecule, consistent with the presence of a hydroxyl substituent ([C_23_H_28_N_2_O_4_+H]^+^, mass error: 1 ppm, Figure 3A).^24^ MGP displayed characteristic fragment ions at *m*/*z* 150.0550 ([C_8_H_7_NO_2_+H]^+^, mass error: −0.7 ppm) assigned as an even-electron fragment formed through rearrangement-assisted cleavage of the indole-containing moiety, and at *m*/*z* 383.1965 ([C_22_H_26_N_2_O_4_+H]^+^, mass error: 0.3 ppm) via the neutral loss of methanol from the methyl ester group, respectively (Figures 2C and 3B). In contrast, the partially coeluting peak at 3.49 min produced a distinct fragment at *m*/*z* 185.0835 (Figure 2D), while the peak at 3.99 min exhibited fragmentation resembling MGP but with different retention behavior and relative ion intensity ratios (Figure 2E). Selected reaction monitoring (SRM) transitions targeting unique fragment ions enabled complete differentiation of MGP from the partially coeluting 3.49 min peak. These findings demonstrate that accurate mass measurement and monitoring of a single fragment ion are insufficient for unambiguous identification in complex matrices.

**Figure 3:**
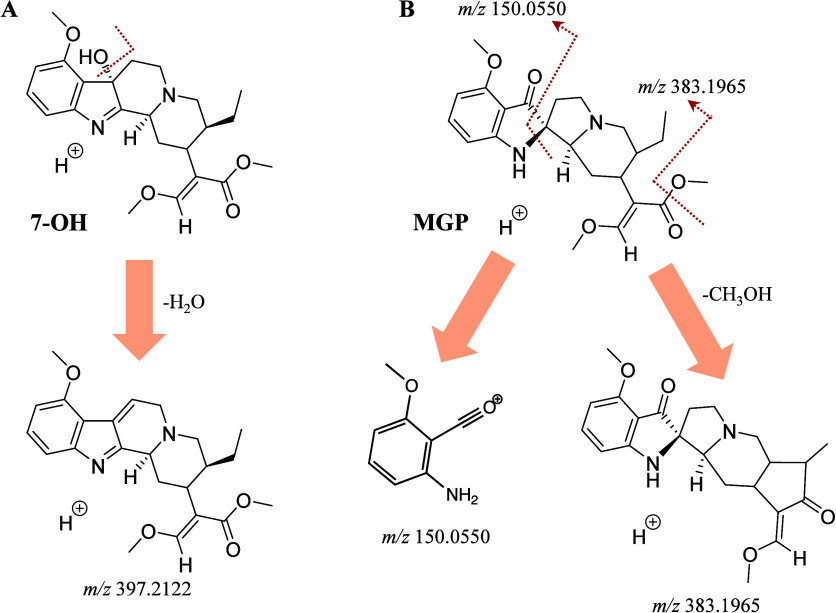
Formation of characteristic ions, including *m*/*z* 397.2122 for 7-OH (A) and *m*/*z* 150.0550 and *m*/*z* 383.1965 for MGP (B) in the MS2 spectrum via HCD.

Importantly, much of the existing LC–MS/MS literature has monitored 7-OH using the transition *m*/*z* 415 → 190, as *m*/*z* 190 is frequently reported as a high-abundance product ion.^25–27^ While *m*/*z* 190 is indeed the most intense fragment for 7-OH in our experiment, it is not structurally unique and may be produced by other closely related alkaloid isomers in kratom extracts. The transition *m*/*z* 415 → 190 provides no additional specificity to differentiate 7-OH/MGP alkaloid isomers, as all the peaks from EIC of *m*/*z* 415.2227 in Figure 2A are also observed in Figure 2F.

For the authentic leaf extract (KM40), a peak at 2.40 min was observed in the EIC at *m*/*z* 415.2227 (Figure 2G), which exactly matched the 7-OH retention time. Even when the *m*/*z* 415 → 190 transition was evaluated, a peak at approximately 2.4 min was still present in the authentic leaf extract (Figure 2L). However, this peak did not meet the defined confirmation criteria established with authentic standards, including retention-time matching and the presence of characteristic secondary fragment ions, such as *m*/*z* 397.2126 (Figure 2H). If identification had relied solely on the high-resolution mass accuracy of *m*/*z* 415.2227 and/or uncharacteristic *m*/*z* 415 → 190 transition, even when retention time matched that of the authentic standard, this peak could have been misassigned as 7-OH, resulting in a false positive identification. These observations demonstrate that reliance solely on the *m*/*z* 415 → 190 transition and retention time matching, which has been overwhelmingly used in the existing literature, can lead to overestimation or misidentification of 7-OH in crude leaf matrices. Incorporation of chromatographic resolution, high-resolution mass accuracy, and secondary diagnostic fragment ions substantially increases analytical specificity and reduces the likelihood of false positive assignments.

The exclusion of MGP in the authentic leaf extract samples was confirmed by the absence of a chromatographic peak at 3.63 min under its characteristic *m*/*z* 415 → 383 transition (Figure 2I). Similarly, both compounds corresponding to the peaks at 3.49 and 3.99 min in the tablet sample (Figure 2D,E) were confirmed absent in the authentic leaf extract (Figure 2J,K). In addition, the peak intensities at *m*/*z* 415.2227 for the authentic leaf extract were approximately 250-fold lower in intensity than those observed in the 7-OH tablet extract. None of these peaks matched both the retention time of authentic 7-OH or MGP and the diagnostic fragment ions required for confirmation. These findings indicate that, under rigorous chromatographic and MS/MS confirmation criteria, 7-OH and MGP were not detected in the analyzed authentic leaf sample. While mitragynine was identified in all 39 samples, 7-OH was confirmed present in 3 capsules and all 9 tablet samples, and 8 out of 9 tablets and 2 capsules contained MGP. The combined use of retention time matching, accurate mass measurement, and diagnostic product ions is necessary in both confirming presence and avoiding false-positive identifications.

To our knowledge, this study represents the first systematic demonstration of the potential for false-positive identification of 7-OH in crude kratom matrices when relying solely on commonly monitored transitions, such as *m*/*z* 415 → 190, and retention-time matching. The analytical framework presented here provides improved specificity for distinguishing confirmed 7-OH and MGP from isomeric and coeluting interferences in complex botanical samples and consumable products. The proposed multicriteria confirmation strategy consists of (i) retention time matching with authentic standards, (ii) accurate mass measurement, and (iii) the presence of multiple diagnostic fragment ions. While accurate mass measurement is enabled by HRMS instrumentation, the identification specificity in this workflow is primarily driven by chromatographic resolution and structurally informative MS/MS fragments (e.g., *m*/*z* 397.2122 for 7-OH and *m*/*z* 150.0550 and 383.1965 for MGP). These fragment ions can be monitored using SRM transitions on triple quadrupole instruments, enabling implementation of this strategy in routine analytical laboratories without HRMS capability. The recommended confirmation criteria for 7-OH and MGP are summarized in Table 1. This broadens the practical applicability of the approach for laboratories performing regulatory, forensic, or quality control testing of botanical products.

**Table 1:** Confirmation Criteria for 7-OH and MGP

compound	RT (min)	precursor (*m*/*z*)	diagnostic fragments (*m*/*z*)	criteria
7-OH	2.40	415	397	RT + fragment
MGP	3.63	415	150 or 383	RT + fragment

### Multivariate Metabolomic Profiling and Sample Classification

To complement the targeted alkaloid analysis, a metabolomic workflow was applied to evaluate whether overall metabolite feature distributions could distinguish vegetation leaf powders and powder extracts, including the authentic leaf materials, from commercial kratom products containing 7-OH and related constituents. High-resolution full-scan LC–MS data were processed to extract molecular features across all samples, followed by alignment, normalization, and filtering to remove background and low-reproducibility signals. PCA of the aligned feature matrix revealed clear separation between authentic leaf materials and 7-OH-enriched commercial products (Figure 4A). PC1 and PC2 explained 74.8% and 15.7% of the total variance, respectively. The authentic leaf samples clustered tightly and overlapped with commercial kratom herb extracts and powder samples, indicating relatively consistent metabolomic profiles characterized by a complex distribution of alkaloids and nonalkaloid phytochemicals. In contrast, the 7-OH tablet samples formed a distinct cluster, reflecting a simplified compositional profile dominated by mitragynine and 7-OH, with reduced representation of minor alkaloids and other secondary metabolites. The gummy formulation samples occupied an intermediate position in the score plot. While lacking detectable 7-OH under validated confirmation criteria, their metabolite profiles differed from authentic leaf materials, consistent with processing, formulation additives, and potential selective extraction of alkaloids. Loading analysis (Figure 4B) indicated that features corresponding to natural indole alkaloids such as MG, speciociliatine, and paynantheine isomer, contributed strongly to the separation of authentic leaf samples, whereas elevated 7-OH intensity was a primary contributor to the differentiation of tablet products. These findings demonstrate that multivariate metabolomic profiling provides complementary evidence supporting the compositional distinctions observed in targeted analyses.

**Figure 4:**
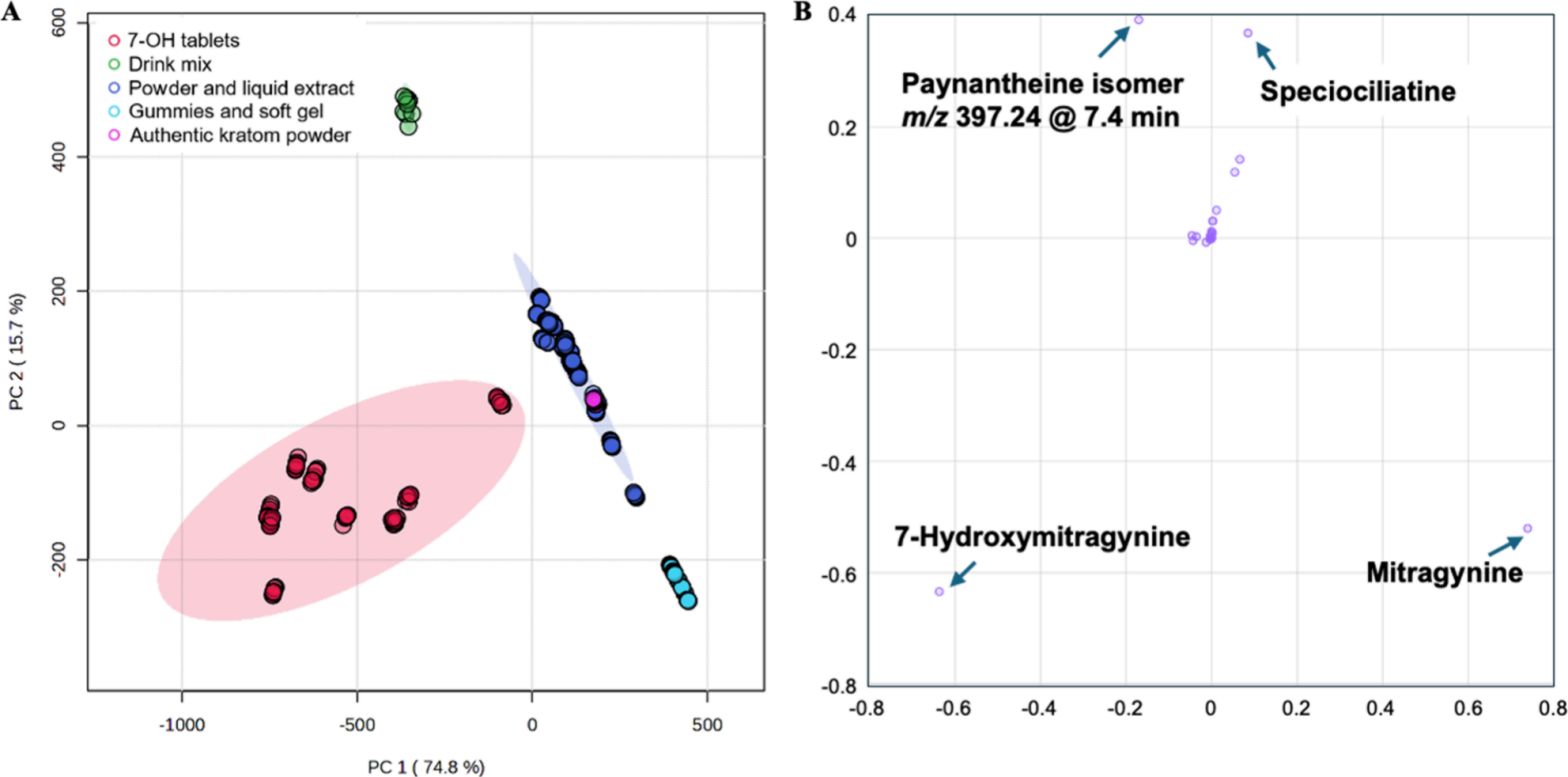
PCA score plot (A) and loading plot (B) for the kratom products based on 350 LC–MS runs.

To further characterize compositional variability, selected metabolite features were examined using box plot visualization across sample groups (Figure 5). Authentic leaf along with kratom herb extracts and powder samples displayed a broader distribution of minor alkaloid features, including diastereomeric compounds at *m*/*z* 399.2284 and related indole derivatives. In contrast, 7-OH tablet products exhibited significantly elevated relative abundance of features corresponding to 7-OH, with reduced variability among other minor alkaloids. This compositional contraction is consistent with selective enrichment or concentration of specific alkaloids relative to the native plant matrix. The gummy products showed lower total feature counts and reduced abundance of nonalkaloid phytochemicals, consistent with removal or degradation during processing and formulation. Importantly, features contributing most strongly to group separation were not limited to 7-OH alone. Rather, classification was driven by the combined distribution of multiple metabolite features (i.e., epicatechin), indicating that global compositional patterns provide a more robust framework for differentiation than reliance on a single marker compound. These results demonstrate that untargeted metabolomic profiling provides orthogonal evidence supporting the targeted LC–MS identification results.

**Figure 5:**
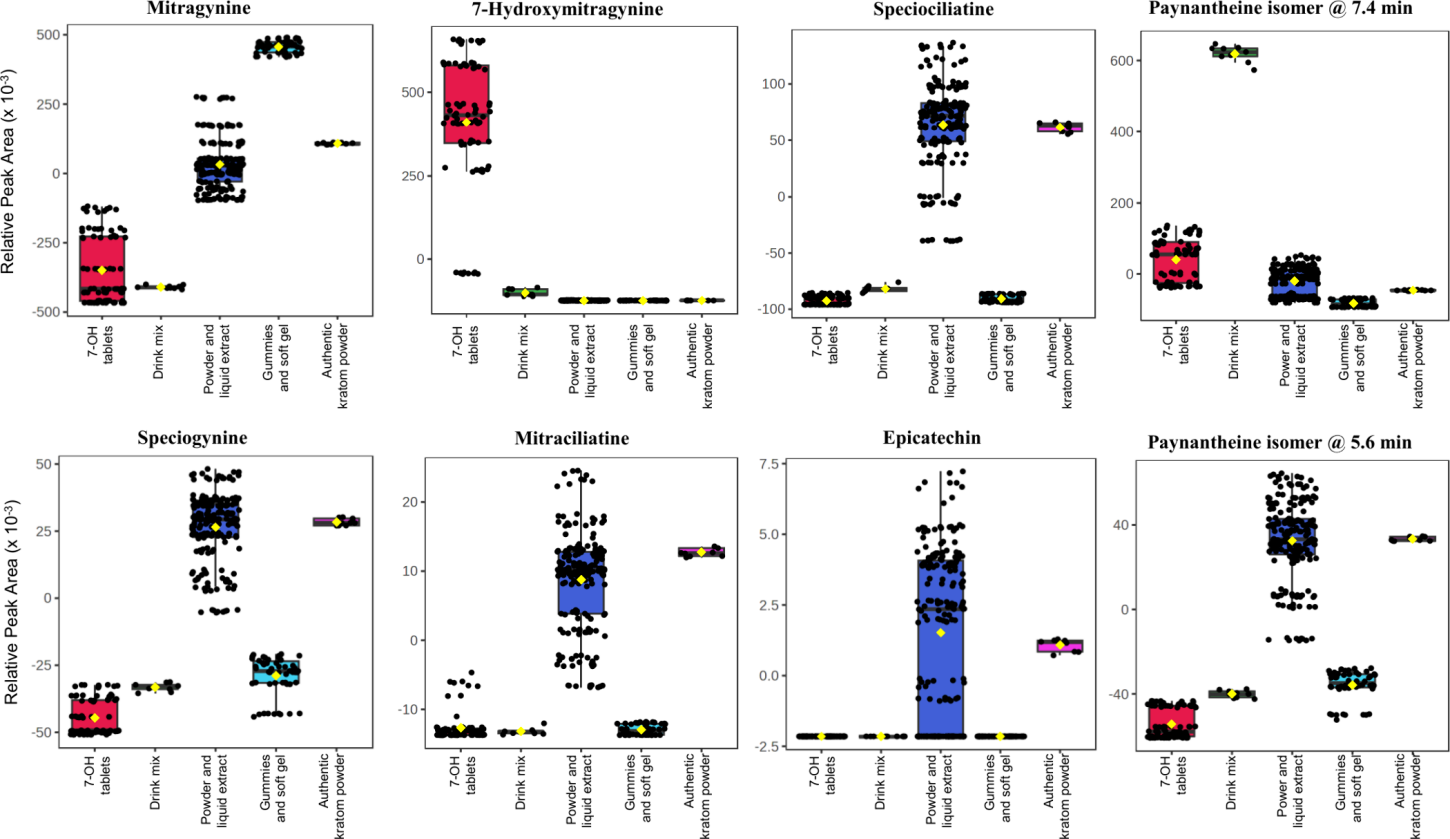
Relative peak areas of discriminative features for various groups of kratom samples. The peak areas were normalized to the sum of all the feature peak areas and then mean-centered.

### Semiquantitative Analysis of 7-OH, MGP, and MG in the Samples

Semiquantitative analysis of MG, 7-OH, and MGP was performed using external calibration curves constructed for each analyte within the dynamic range of 0.1–5 μg mL^–1^. Calibration curves for MG, 7-OH, and MGP exhibited good linearity with representative R^2^ values exceeding 0.995 (Figure S2). These results indicate acceptable analytical performance for semiquantitative comparison across samples. The limits of detection (LOD) were determined by serial dilution of the standards until the signal intensity reached three standard deviations above the baseline noise. The LODs were estimated to be 1 ng mL^–1^ for 7-OH, 0.5 ng mL^–1^ for MGP, and 1 ng mL^–1^ for MG. The limit of quantitation (LOQ) was defined as the lowest calibration concentration (0.1 μg mL^–1^), for which signal-to-noise ratios were substantially greater than 10, ensuring reliable quantification.

The concentrations of individual alkaloids in the samples were first calculated in units of mg g^–1^ using the following relationship:

$$\frac{P_{0}-b_{0}}{m_{0}}\times \frac{V}{W}\times \frac{D}{100}$$

where P_0_ represents the peak area of the target analyte in the sample chromatogram, b_0_ and m_0_ correspond to the intercept and slope of the calibration curve, respectively, V is the volume of the extracted solution (mL), W is the sample weight (g), and D represents the dilution factor. The calculated concentrations were subsequently converted to mg per serving using the serving size specified on the product label.

Among the 9 tablet products analyzed, 7-OH was detected in 7 samples, and approximately half of these contained levels 20–79% lower than those declared on the product labels (Table S2). Values in Table S2 reported as N.D. indicate concentrations below LOD, while N.Q. indicates concentrations detected but below LOQ. In addition, three capsule products (KM24, KM25, and KM26) were found to contain detectable levels of 7-OH. Notably, one capsule product (KM25) labeled as “made from 100% pure kratom leaves” contained 0.14 ± 0.03 mg per serving of 7-OH, whereas the remaining two capsule samples contained trace levels that were more than 10-fold below LOQ, preventing reliable quantification. Notably, detectable 7-OH was observed almost exclusively in tablet formulations, whereas plant powder, liquid extracts, gummies, and most capsule products contained no detectable 7-OH. This distribution further supports the hypothesis that highly processed formulations may involve enrichment or chemical modification of kratom alkaloids relative to the native plant matrix.

MGP was labeled on only one product (tablet sample KM03), yet it was detected in seven additional tablet products and two capsule products. In KM03, the measured MGP concentration was 3.8 ± 0.4 mg per serving, compared with a labeled value of 7.5 mg per serving. Three other tablet products (KM05, KM06, and KM08) contained 0.24 ± 0.01, 0.20 ± 0.01, and 0.28 ± 0.01 mg per serving, respectively. In the remaining products in which MGP was detected, concentrations were below the LOQ but were still confidently confirmed using the multitier identification criteria described earlier. These findings suggest that MGP may be more widely present in commercial kratom products than indicated by product labeling, though there is no requirement to list constituent profiles.

The MG content exhibited substantial variability across commercial products, ranging from not quantifiable (<LOQ) to 312 mg per serving (Table S2), whereas the authentic leaf-cut sample contained 6.3 mg g^–1^ of MG. Only one tablet product (KM03) reported an MG content on the label (7.5 mg per serving), and the measured value was consistent with this claim at 8.4 ± 0.4 mg per serving. In contrast, MG concentrations in the other 8 tablet samples were much lower, ranging from not quantifiable to 1.71 mg per serving, although their labels did not specify MG content. In contrast to the tablet products, which generally contained very low levels of MG, liquid extracts, gummies, and gel formulations exhibited substantially higher MG concentrations, in several cases exceeding 100 mg per serving. This variability indicates large differences in extraction efficiency, formulation concentration, and potential product standardization across commercial kratom products. In several products, particularly concentrated extract and gummy formulations, the measured MG content substantially exceeded the labeled amount (e.g., KM14, KM16, KM28, KM29, and KM30), suggesting inconsistent manufacturing practices or inaccurate labeling of kratom alkaloid content.

Unit-to-unit variability was assessed by analyzing five individual tablets or capsules and expressed as relative standard deviation (RSD), and the results are summarized in Table S3. The 7-OH content showed moderate tablet-to-tablet variability, with RSD values ranging from 10–17% (n = 5). In contrast, MGP exhibited greater variability, with RSD values of 24–27%. The variability of MG in capsule products was product-dependent, with one product showing relatively low variability (10% RSD) and another showing substantially higher variability (48% RSD). Overall, these results highlight significant heterogeneity in alkaloid content among individual units, particularly for MGP and certain capsule formulations, which may have implications for dosing consistency and potential toxicological risks in consumer use. It should be noted that the concentrations reported here may represent conservative estimates, as external calibration without matrix-matched standards can lead to underestimation due to ion suppression effects during LC–MS analysis. The quantitative results reported here should therefore be interpreted as semiquantitative estimates intended to evaluate relative product composition rather than validated regulatory measurements. In complex dietary supplement matrices such as gummies, significant ion suppression has previously been observed, with reductions of up to 58% and 38% for tetrahydrocannabinol (THC) and tetrahydrocannabinolic acid (THCA), respectively, in cannabis products in comparable analytical systems.^18^ Future studies will incorporate stable-isotope-labeled internal standards to improve quantitative accuracy and compensate for matrix effects. Taken together, the semiquantitative results demonstrate that commercial kratom products vary widely in alkaloid composition, both in absolute concentrations and in the relative distribution of MG, 7-OH, and MGP. This variability highlights the lack of standardization in the commercial marketplace and reinforces the need for robust analytical methods for product characterization and regulatory oversight.

Despite these analytical limitations, the detection of 7-OH at concentrations exceeding labeled values is noteworthy given the compound’s ambiguous regulatory status across different U.S. jurisdictions. Similarly, the detection of MGP, even at trace levels, in products where it is not declared on the label raises concerns regarding product authenticity and manufacturing practices. MGP is known to possess significantly higher opioid receptor potency than morphine and is generally considered to arise from metabolic transformation or synthetic processes rather than natural biosynthesis in the plant.

These findings highlight the potential importance of quantitative determination of 7-OH and MGP in regulatory and forensic contexts. For example, the emergency rule issued under Ohio Administrative Code (OAC) 4729:9–1–01.1 in December 2025 classified synthetic kratom-related products as Schedule I controlled substances, while allowing kratom sold in its natural vegetation state (crude leaf products), provided that only trace levels of 7-OH are present.^28^ However, the regulatory definition of “trace amounts” remains unclear. As kratom regulations continue to evolve, robust analytical methods capable of reliably identifying and quantifying potent alkaloids will be essential for law enforcement, regulatory oversight, and public health protection.

In addition to regulatory applications, the analytical framework described here may also support standardization of kratom botanical materials and pharmacological investigations, including studies on the metabolism and biological effects of kratom alkaloids.

## Conclusion

This study provides a comprehensive LC–MS framework for the reliable identification and quantification of key kratom alkaloids in commercial products and botanical materials. Through systematic evaluation of chromatographic behavior and MS/MS fragmentation patterns, we demonstrate that reliance on commonly used transitions, particularly *m*/*z* 415 → 190, can lead to false-positive identification of 7-OH in complex kratom matrices. The integration of chromatographic resolution, accurate mass measurement, and diagnostic fragment ions enables confident differentiation of structurally related alkaloids, including MG, 7-OH, MGP, and closely related isomers. These findings highlight the importance of multicriteria LC**–**MS confirmation strategies when analyzing complex botanical products.

Application of this analytical workflow to a diverse set of commercial kratom products revealed substantial variability in alkaloid composition and identified multiple products containing 7-OH and MGP, including cases where these compounds were not disclosed on product labels. The quantitative results further demonstrate wide variation in MG content among products, underscoring the lack of standardization across the marketplace. Together, these observations illustrate how LC**–**MS can provide both targeted confirmation and broader compositional profiling for emerging botanical products. The multitier identification strategy presented in this work improves analytical specificity and may support regulatory testing, forensic investigations, and quality control of botanical materials. Because the confirmation strategy relies primarily on chromatographic separation and diagnostic fragmentation patterns rather than high-resolution mass measurements, the approach can be readily implemented on widely available tandem mass spectrometry platforms. As regulatory scrutiny of kratom and related products continues to evolve, robust MS-based analytical methods will play an increasingly important role in ensuring product authenticity, safety, and accurate chemical characterization. Future studies will focus on developing and validating robust quantitative LC–MS methods for the accurate determination of key kratom alkaloids in commercial products and botanical materials.

## Supplementary Material



## Notes

Tables S1–S3: Kratom-related products information; Semiquantitative results for the 7-OH, MGP, and MG in the kratom-related products; Unit-to-unit variability of 7-OH, MGP, and MG in selected kratom products. Figure S1–S2: EIC of *m*/*z* 399.2284 (A), *m*/*z* 415.2227 (B), *m*/*z* 397.2122 (C), and *m*/*z* 291.0863 (D) for the authentic kratom sample (K-55B) reconstructed from raw LC–MS data previously published by Cech’s group; Representative calibration curves for MG (A), 7-OH (B), and MGP (C) (n = 3) (PDF)
