# Supplementary material for: Avoiding False Identification of 7‑Hydroxymitragynine in Kratom Products Using a Multicriteria LC–MS Confirmation
Source: J Am Soc Mass Spectrom. 2026 Apr 16;37(5):1303–12. doi: 10.1021/jasms.6c00088 (PMC13154205; doi:10.1021/jasms.6c00088)
Supplement: Supplementary file 1 [file js6c00088_si_001.pdf]

Supplemental Material

Avoiding False Identification of 7-Hydroxymitragynine in Kratom Products Using a Multi-Criteria LC–MS Confirmation

Daniel Sheehan[1], Yanfang Li[1], Emily Meckler[1], Roy Upton[2], Mengliang Zhang[1,*]

[1]Department of Chemistry and Biochemistry, Ohio University, Athens, OH, 45701

[2]American Herbal Pharmacopoeia, Scotts Valley, CA 95066

*Corresponding authors: Mengliang Zhang, *E-mail address:* zhangm4@ohio.edu. Phone: +01 740-593-0719

Table S1. Summary of products analyzed in this study

ID	Product Name	Product Type	Source
KM01	Brand 1 Product 1	Tablet	Online
KM02	Brand 2 Product 1	Tablet	Online
KM03	Brand 3 Product 1	Tablet	Online
KM04	Brand 4 Product 1	Tablet	Online
KM05	Brand 5 Product 1	Tablet	Online
KM06	Brand 6 Product 1	Tablet	Online
KM07	Brand 2 Product 2	Tablet	Online
KM08	Brand 3 Product 2	Tablet	Online
KM09	Brand 7 Product 1	Drink Mix	Online
KM10	Brand 8 Product 1	Powder	Online
KM11	Brand 9 Product 1	Capsule	Online
KM12	Brand 10 Product 1	Capsule	Online
KM13	Brand 11 Product 1	Capsule	Online
KM14	Brand 12 Product 1	Gel	Online
KM15	Brand 13 Product 1	Liquid	Online
KM16	Brand 14 Product 1	Gel	Online
KM17	Brand 15 Product 1	Powder	Online
KM18	Brand 16 Product 1	Tablet	Online
KM19	Brand 14 Product 2	Liquid	Online
KM20	Brand 12 Product 2	Liquid	Online
KM21	Brand 10 Product 2	Liquid	Online
KM22	Brand 17 Product 1	Liquid	Online
KM23	Brand 18 Product 1	Liquid	Online
KM24	Brand 10 Product 3	Capsule	Online
KM25	Brand 10 Product 4	Capsule	Online
KM26	Brand 19 Product 1	Capsule	Online
KM27	Brand 20 Product 1	Gummy	Online
KM28	Brand 21 Product 1	Gummy	Online
KM29	Brand 22 Product 1	Gummy	Online
KM30	Brand 23 Product 1	Gummy	Online
KM31	Brand 24 Product 1	Capsule	Online
KM32	Brand 10 Product 5	Capsule	Online
KM33	Brand 25 Product 1	Capsule	Online
KM34	Brand 25 Product 2	Capsule	Online
KM35	Brand 26 Product 1 (Excluded)	Liquid	Local
KM36	Brand 8 Product 2	Powder	Local
KM37	Brand 8 Product 3	Powder	Local
KM38	Brand 8 Product 4	Powder	Local
KM39	Brand 26 Product 1	Powder	Online
KM40	Authenticated kratom voucher specimen (AHP-Verified)	Powder	AHP

Table S2. Semi-quantitative results for the 7-OH, MGP, and MG in the kratom-related products

	Serving Size	Calculated 7-OH (mg/Serving)	Claimed 7-OH (mg/Serving)	Calculated MGP (mg/Serving)	Claimed MGP (mg/Serving)	Calculated MG (mg/Serving)	Claimed MG (mg/Serving)
KM01	½ tablet	5.5 ± 0.1	9	N.D.	N.A.	0.18 ± 0.01	N.A.
KM02	½ tablet	3.9 ± 0.2	7.5	N.Q.	N.A.	N.Q.	N.A.
KM03	½ tablet	4.2 ± 0.2	7.5	3.8 ± 0.4	7.5	8.4 ± 0.4	7.5
KM04	½ tablet	7.3 ± 0.2	9	N.Q.	N.A.	0.20 ± 0.02	N.A.
KM05	½ tablet	5.2 ± 0.1	8	0.24 ± 0.01	N.A.	N.Q.	N.A.
KM06	½ tablet	7.8 ± 0.2	10	0.20 ± 0.01	N.A.	0.82 ± 0.05	N.A.
KM07	½ tablet	6.0 ± 0.3	7.5	N.Q.	N.A.	N.Q.	N.A.
KM08	½ tablet	6.0 ± 0.1	9	0.28 ± 0.01	N.A.	1.71 ± 0.04	N.A.
KM09	5 g	N.D.	N.A.	N.D.	N.A.	N.Q.	N.A.
KM10	0.6 g	N.D.	N.A.	N.D.	N.A.	10.5 ± 0.2	≤ 9.1
KM11	1 capsule	N.D.	N.A.	N.D.	N.A.	6.33 ± 0.07	7.8
KM12	1 capsule	N.D.	N.A.	N.D.	N.A.	8.3 ± 0.3	N.A.
KM13	1 capsule	N.D.	N.A.	N.D.	N.A.	5.90 ± 0.06	N.A.
KM14	6.5 g	N.D.	N.A.	N.D.	N.A.	123 ± 26	50.4
KM15	14 mL	N.D.	N.A.	N.D.	N.A.	16 ± 1	N.A.
KM16	4.8 g	N.D.	N.A.	N.D.	N.A.	312 ± 43	50
KM17	3 g	N.D.	N.A.	N.D.	N.A.	24 ± 6	N.A.
KM18	½ tablet	3.1 ± 0.1	15	N.Q.	N.A.	0.54 ± 0.07	N.A.
KM19	3 mL	N.D.	N.A.	N.D.	N.A.	33 ± 2	25
KM20	15 mL	N.D.	N.A.	N.D.	N.A.	67 ± 3	95
KM21	10 mL	N.D.	N.A.	N.D.	N.A.	90 ± 4	100
KM22	60 mL	N.D.	N.A.	N.D.	N.A.	41 ± 1	65
KM23	30 mL	N.D.	N.A.	N.D.	N.A.	16 ± 5	37.5
KM24	1 capsule	N.Q.	N.A.	N.D.	N.A.	33 ± 2	8.65
KM25	1 capsule	0.14 ± 0.03	N.A.	N.Q.	N.A.	58 ± 7	90
KM26	1 capsule	N.Q.	N.A.	N.Q.	N.A.	17 ± 1	85
KM27	1 softgel	N.D.	N.A.	N.D.	N.A.	41 ± 5	45
KM28	1 gummy	N.D.	N.A.	N.D.	N.A.	114 ± 12	25
KM29	1 gummy	N.D.	N.A.	N.D.	N.A.	275 ± 87	50
KM30	1 gummy	N.D.	N.A.	N.D.	N.A.	218 ± 24	35
KM31	1 capsule	N.D.	N.A.	N.D.	N.A.	5 ± 1	≤ 9.3
KM32	3 capsules	N.D.	N.A.	N.D.	N.A.	15 ± 2	N.A.
KM33	6 capsules	N.D.	N.A.	N.D.	N.A.	36 ± 5	N.A.
KM34	6 capsules	N.D.	N.A.	N.D.	N.A.	30 ± 1	N.A.
KM36	0.6 g	N.D.	N.A.	N.D.	N.A.	3.8 ± 0.3	N.A.
KM37	0.6 g	N.D.	N.A.	N.D.	N.A.	5.4 ± 0.5	N.A.
KM38	0.6 g	N.D.	N.A.	N.D.	N.A.	3.8 ± 0.1	N.A.

*N.A.: information not available; N.D.: Not detected (below LOD); N.Q.: Not quantifiable (below LOQ)

Table S3. Unit-to-unit variability of 7-OH, MGP, and MG in selected kratom products

	Serving Size	Calculated 7-OH (mg/Serving)	7-OH Variability (%)	Calculated MGP (mg/Serving)	MGP Variability (%)	Calculated MG (mg/Serving)	MG Variability (%)
KM05	½ tablet	4.7 ± 0.8	17	0.26 ± 0.07	27	N.Q.	N.A.
KM06	½ tablet	7.4 ± 1	14	0.22 ± 0.05	24	0.88 ± 0.12	13
KM18	½ tablet	3.4 ± 0.4	10	N.Q.	N.A.	0.94 ± 0.09	9
KM31	1 capsule	N.D.	N.A.	N.D.	N.A.	8.9 ± 0.9	10
KM33	6 capsule	N.D.	N.A.	N.D.	N.A.	20 ± 10	48

*N.A.: information not available; N.D.: Not detected (below LOD); N.Q.: Not Quantifiable (below LOQ); Variability was assessed by analyzing five (n = 5) individual tablets or capsules and expressed as relative standard deviation (RSD).

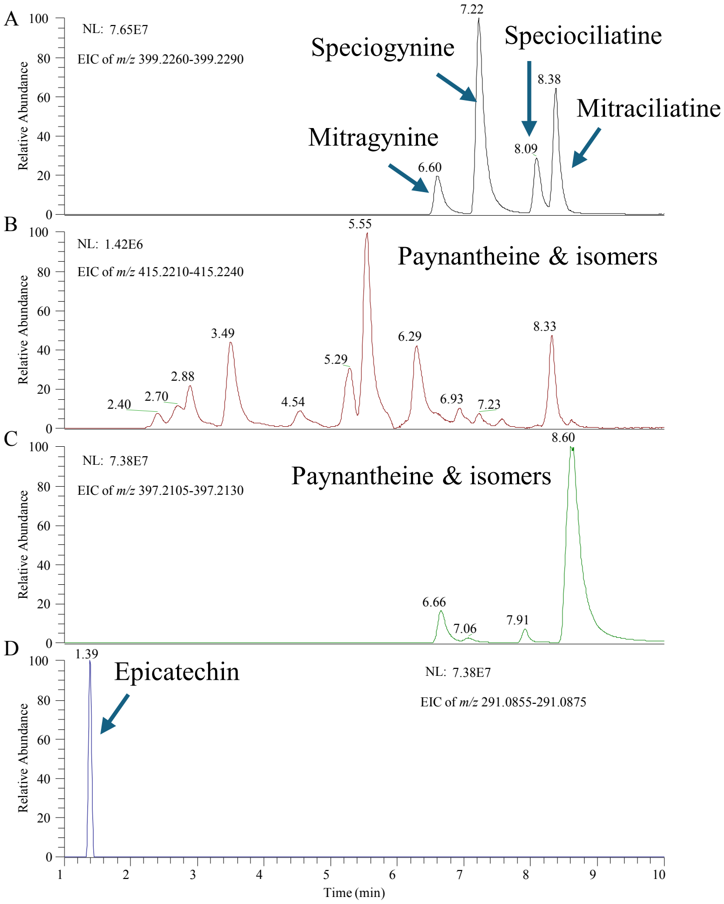

Figure S1. EIC of *m/z* 399.2284 (A), *m/z* 415.2227 (B), *m/z* 397.2122 (C), and *m/z* 291.0863 (D) for the authentic kratom sample (K-55B) reconstructed from raw LC-MS data previously published by Cech's group.[22]

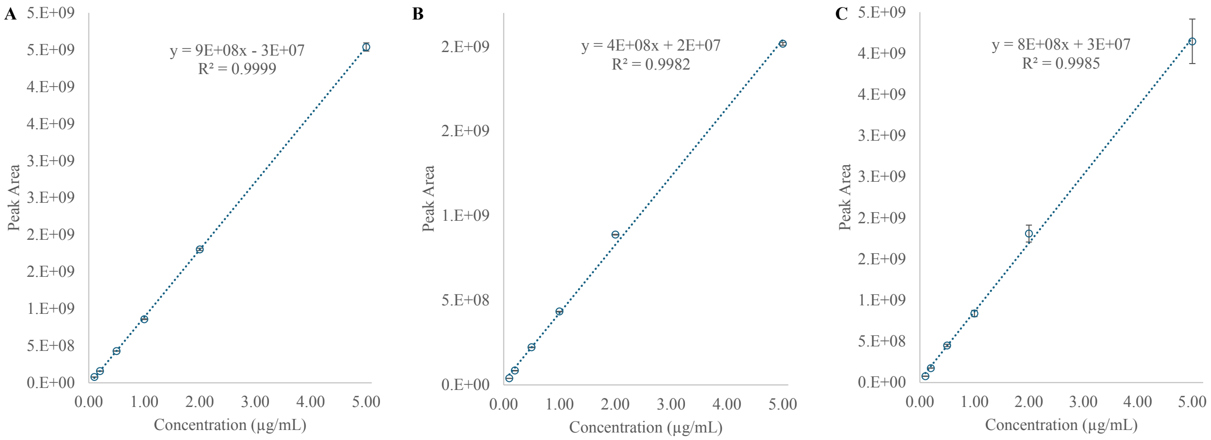

Figure S2. Representative calibration curves for MG (A), 7-OH (B), and MGP (C) (n = 3).
